# Misjudging early embryo mortality in natural human reproduction

**DOI:** 10.12688/f1000research.22655.1

**Published:** 2020-07-14

**Authors:** Gavin E. Jarvis

**Affiliations:** 1 Department of Physiology, Development and Neuroscience, University of Cambridge, Cambridge, CB2 3EG, UK

**Keywords:** Mr Justice Munby, Smeaton, morning-after pill, embryo mortality, early pregnancy loss

## Abstract

In 2002, in a judgment relating to the use of the morning-after pill, Mr Justice Munby held that pregnancy begins with the implantation of an embryo into the uterus of a woman. The case involved a large body of expert witness evidence including medical and physiological details of human reproduction. Munby J. emphasised one particular aspect of this evidence: namely, the developmental failure rate of human embryos after fertilisation. Under natural conditions, embryo loss is approximately 10-40% before implantation, and total loss from fertilisation to birth is 40-60% (Jarvis, 2016). By contrast, and based on expert witness testimony, Munby J. stated that not much more than 25% of successfully fertilised eggs reach the implantation stage, and that fewer than 15% of fertilised eggs result in a birth, figures that do not accurately represent scientific knowledge regarding human embryo mortality and pregnancy loss under natural conditions. Rather, these figures were derived from experimental laboratory data and clinical outcomes from *in vitro* fertilisation treatment. Testimony provided by other expert witnesses directly contradicted these specific numerical claims. In emphasising these figures, Munby J. gave the impression that human embryo mortality is substantially higher than available scientific evidence indicated. In this critique, all the scientific expert witness evidence is presented and reviewed, and an explanation provided for why the emphasised figures are wrong. Whether there are implications of Munby J.’s scientific misjudgment on the legal outcome is for others to consider.

## Introduction

In 2002, a judicial review was considered by the Honourable Mr Justice Munby of the Queen’s Bench Division (Administrative Court) to determine whether the supply of Levonelle (commonly referred to as a morning-after pill (MAP)) by pharmacists amounted, in principle, to a criminal act under section 58 and/or section 59 of the Offences against the Person Act 1861 ^1, a^. Counsel for the claimant argued that the MAP could act by preventing the implantation of a fertilised egg in the uterus and that its use for this purpose therefore constituted an act intended to bring about a miscarriage. Counsel argued that the supply and use of the MAP for this purpose ought therefore to be regulated in the same way as surgically- or medically-induced abortion, as required by the Abortion Act 1967. A judgment in favour of the claimant would have had a significant social impact on the supply of the MAP.

The Claimant was John Smeaton, on behalf of the Society for the Protection of Unborn Children (SPUC). The Defendant was The Secretary of State for Health, and the two Interested Parties were Schering Health Care Ltd and the Family Planning Association.

Munby J. held that “the prescription, supply, administration or use of the morning-after pill does not – indeed cannot – involve the commission of any offence under either section 58 or section 59 of the 1861 Act” ^b^.

The case included evidence from scientific expert witnesses and in his judgment, which Munby J. conceded was “necessarily very long” ^c^, he described and scrutinised aspects of this evidence in detail.

The primary purpose of this article is to shed light upon one aspect of the scientific expert witness evidence, uncritically accepted and deliberately emphasised by Munby J., which was inaccurate and glaringly inconsistent: namely, the extent of natural embryo mortality in humans in the first week after fertilisation. The nature of the error, the inconsistencies in the expert evidence, and their sources are reviewed and explained.

Whether any legal implications arise from the inconsistent expert witness testimony and judicial error is for others to consider.

## Expert witness statements

Copies of original expert witness statements used in this critique were made available freely on request from the archives of the Claimant. Civil Procedure Rules concerning the use of witness statements for other purposes indicate that where a witness statement has been put in evidence at a hearing held in public, its use is not restricted to the purpose of the proceedings in which it is served ^d^. Regarding subsequent use of disclosed documents: they may be used only for the purpose of the proceedings in which they are disclosed, except where they have been read to or by the court, or referred to, at a hearing that has been held in public ^e^. The substantive hearing was held in public and lasted three days, starting on 12 ^th^ February 2002 ^f^. All witness statements referred to in this article were read and referred to by Munby J. No court order has been made restricting or prohibiting the use of these expert witness statements. Finally, it is in keeping with principles of open justice (and academic enquiry) that evidence placed before courts be available for public scrutiny, as confirmed in recent case law ^2^.

A full transcript (redacted of personal information) of all these statements is available on request from the author. In producing the transcript from original copies of court papers, every effort has been made to reproduce the content as accurately as possible. Errors and idiosyncrasies in spelling, grammar and style have been retained. Any errors of transcription are entirely the responsibility of this author, and will be corrected on notification. Where consent to publish has been obtained from the witnesses, full transcripts are directly available in the *Underlying data* ^3^ as described in the **Data availability** section. Where consent to publish has not been obtained, only those passages quoted by Munby J. or directly referenced in this article are shown, the remainder being redacted. Transcripts are intended to enable readers to draw their own conclusions about the contents of the judgment and this article.

The scientific and/or medical expert witness statements were provided to the court by eight “very eminent medical experts” ^g^ as summarised in Table 1. Munby J. commended submissions from all parties, both written and oral, as being “uniformly of the very highest quality” ^h^. However, although Munby J. stated that “they were all agreed as to the basic physiology” ^i^, close reading indicates that, with regard to the extent of embryo mortality, this was not the case.

**Table 1.** A summary of expert witnesses, their statements and submissions presented to the Court in R (on the application of Smeaton) v Secretary of State for Health. Word counts for the whole documents and for the sections directly related to embryo loss were obtained from transcripts of the witness statements made into Microsoft Word, and include footnotes. Values are rounded to the nearest 10 words. For the whole published article ( *PB/2*), a sampling strategy was employed to estimate the body text word count and rounded to 100 words. Transcripts of the witness statements are in the *Underlying data.* * The content of Exhibit *PNL1* is available online here: https://www.pharmaceutical-journal.com/learning/learning-article/guidance-on-pharmacy-supply-of-ehc/20003892.article.

Name of witness	Position of witness at time of writing statement	Statement on behalf of	Referred to as	Summary content of statement	Witness statement dated	Total word count	Embryo loss word count
Prof. Chris Barratt	Professor and Head of the Reproductive Biology and Genetics Research Unit at the University of Birmingham	The Claimant: John Smeaton (SPUC)	*WSCB*	The statement describes “the role of sperm in the reproductive process”.	22 October 2001	970	0
Prof. Peter Riven Braude	Professor of Obstetrics and Gynaecology, King’s College London	The Defendant: Secretary of State	*WSPB*	An account of “the human fertilisation and gestation process, up to the point at which the developing embryo has competed its implantation into the wall of the uterus.”	10 July 2001	1,540	270
			Exhibit *PB/2*	“ *The Embryo in Contemporary Medical Science*” by P. R. Braude and M. H. Johnson, from “ *The* *Human Embryo: Aristotle and the Arabic and* *European Traditions*”, edited by G. R. Dunstan, University of Exeter Press, 1990, pp. 208–221.	Published, 1990	Approx. 2,600	140
Prof. Nigel Andrew Brown	Professor of Developmental Biology, St George’s Hospital Medical School, University of London	First Interested Party: Schering Health Care Ltd	*WSNB*	This statement is a “brief description of the first 14 days of the human life cycle, from coitus onwards”.	3 August 2001	3,010	280
Prof. James Owen Drife	Professor of Obstetrics and Gynaecology, School of Medicine, University of Leeds	First Interested Party: Schering Health Care Ltd	*WSJD*	The statement contains, inter alia, definitions of “terms in everyday use in gynaecological practice today”.	3 August 2001	2,570	240
Dr Peter Norman Longthorne	Medical Director of Schering Health Care Ltd	First Interested Party: Schering Health Care Ltd	*WSPL*	The statement provides a description of Levonelle and its mechanism of action.	3 August 2001	2,910	0
			Exhibit *PNL1*	“ *Practice guidance on the supply of Emergency* *Hormonal Contraception as a pharmacy* *medicine.*” This is a guidance document produced by *The* *Royal Pharmaceutical Society*, and published in *The Pharmaceutical Journal*, December 2000, online | URI: 20003892 *	Published, 2000	3,500	0
			Exhibit *PNL2*	Product Information Leaflet for Levonelle™ (Levonorgestrel 750 microgram tablets)	March 2001	1,980	0
Dr John McLean	Formerly a Senior Lecturer in Anatomy and Embryology in the Faculty of Medicine, University of Manchester	The Claimant: John Smeaton (SPUC)	*WSJM1*	The statement is “an account of the first two weeks of human embryogenesis and the incidence of unrecognised early embryo loss”. It includes an opinion on “the possible public health consequences of making emergency hormonal contraception available without the need for medical oversight”.	22 October 2001	6,100	740
			Exhibit *JM1*	Photocopy of schematic figures depicting embryo development from fertilisation to the formation of mesoderm.	22 October 2001	Five figures	0
			*WSJM2*	This additional statement describes “how the commencement of pregnancy is dated”.	4 February 2002	190	0
Dr Connie Smith	A Director at the Clinical Effectiveness Unit at the Faculty of Family Planning and Reproductive Health Care of the Royal College of Obstetricians and Gynaecologists	The Defendant: Secretary of State	*WSCS*	An account of “the action of reversible contraceptive methods”.	12 July 2001	2,480	0
			Exhibit *CS/2*	Figures and tables referred to in the witness statement	12 July 2001	-	-
Prof. Steven Smith	Professor of Obstetrics and Gynaecology and Head of Department in the Department of Obstetrics and Gynaecology at The University of Cambridge	The Claimant: John Smeaton (SPUC)	*WSSS*	An account of the “mode of action of Levonelle”.	19 October 2001	1,950	0

## The incidence of natural human embryo mortality

Before proceeding, it will be helpful to summarise the key biological stages in normal early human reproduction: coitus, ovulation, fertilisation, embryo development and implantation. Munby J. does a commendable job of synthesising the biological evidence in paragraph 126 of his judgment. Coitus introduces sperm into the lower female reproductive tract and ovulation releases an egg into the upper female reproductive tract. Fertilisation occurs in the fallopian tube when sperm and egg meet and one sperm penetrates the egg: “This can be described as Time 0” ^j^. The fertilised egg develops and becomes a blastocyst after 5–6 days. Around day 7, the blastocyst begins to implant into the lining of the uterus. During implantation, the embryo produces human chorionic gonadotrophin (hCG), detection of which “represents the first reliable opportunity to identify the existence of an embryo” ^k^. Approximately 2 weeks after fertilisation, a woman will miss her menstrual period, the first clear external indication of the presence of a developing embryo.

**Figure 1.**
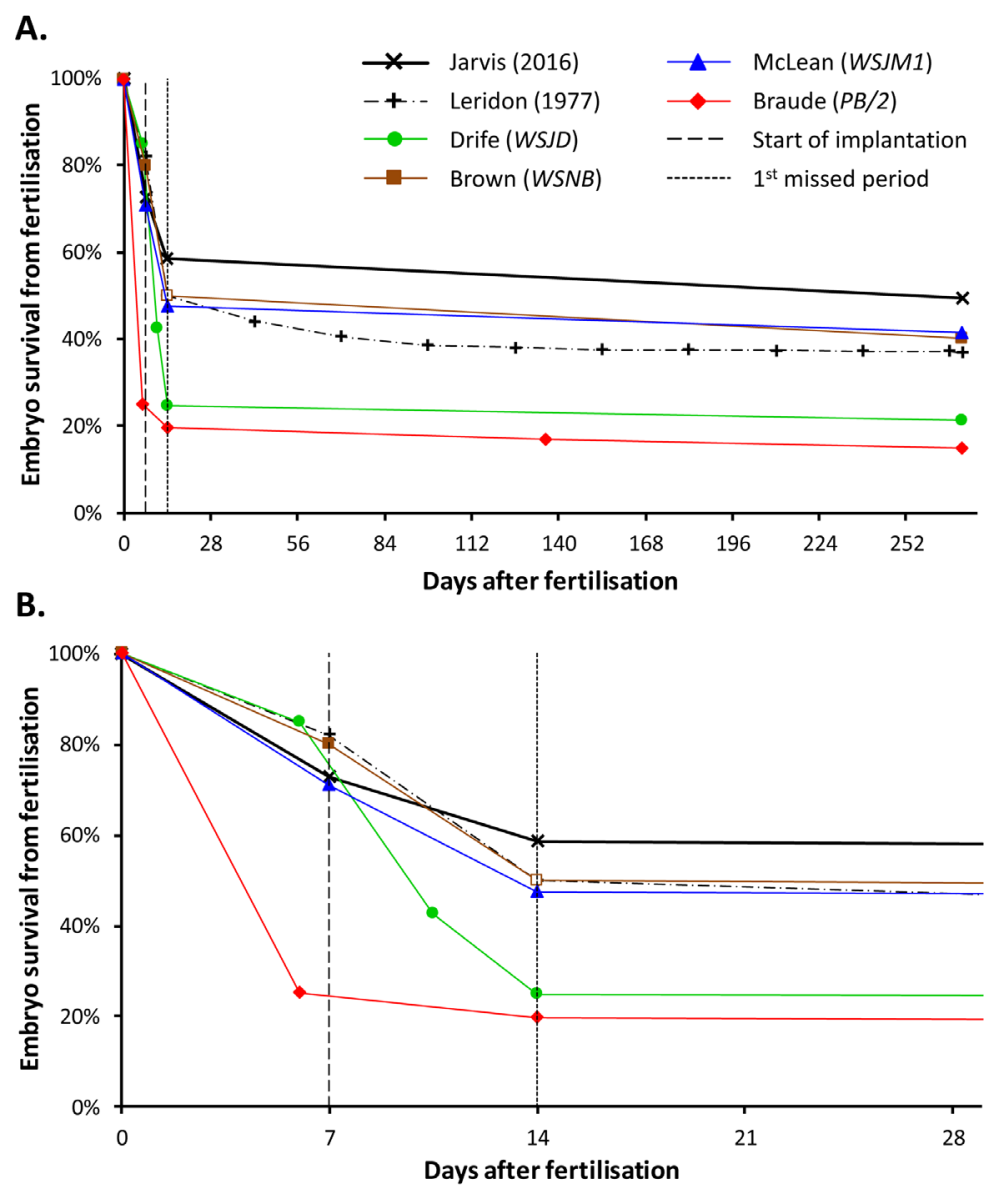
Estimates of embryo survival from fertilisation until ( **A**) birth or ( **B**) four weeks after fertilisation. Numerical values derived directly from witness statements are shown as solid points. Open points have been inferred to facilitate graphical representation. Two sets of reference values have been included for comparison. The first set is derived from Table 3 of Jarvis (2016) by averaging probabilities from three independent studies. The second is from Table 4.20 of Leridon (1977). The figure clearly indicates that the extent of early embryo mortality obtained from Braude ( *PB/2*) and emphasised by Munby J. in his judgment is substantially different from all the other witness statement estimates and those published by Jarvis and Leridon. Drife’s estimate for total pregnancy loss from fertilisation to birth is also excessive compared to the other values. The explanation for the large discrepancy in pre-implantation mortality is that Braude’s estimate is derived from *in vitro* laboratory and clinical IVF data, and not from natural reproduction.

Complex biologic processes do not work perfectly all of the time, including human reproduction. A recent re-analysis has concluded that pre-implantation embryo loss is approximately 10–40% and that total loss from fertilisation to birth is approximately 40–60% ^4^. In addition, a review of scientific data that contribute to quantitative claims regarding natural pregnancy loss provides a detailed background against which claims made by the expert witnesses regarding the incidence of natural human embryo mortality may be evaluated ^5^. Making sense of these numerical estimates is not easy. To aid understanding, Figure 1 summarises conclusions from these articles alongside the claims of the expert witnesses and numerical estimates from an influential and valuable analysis by Henri Leridon ^6^. Details of how the figure was constructed are in the legend.

Four of the eight expert witnesses provided numerical estimates and/or comment on the extent of human embryo mortality: Drife, Brown, Braude and McLean. It is in these quantitative claims regarding embryo mortality that an inconsistency in the evidence and a judicial error of interpretation is apparent. I shall consider each witness statement in turn and then explain the nature and source of the scientific error in Munby J.’s judgment.

### A. Professor James Owen Drife

Professor Drife’s witness statement ( *WSJD*, see *Underlying data*) was quoted verbatim and at length by Munby J. ^l^, including the following section, which summarises numerically the fate of fertilised embryos:

“From various strands of evidence it has been calculated that in a normally cycling woman who is sexually active and not using contraception, conception will occur in about 85% of cycles. Of those fertilised eggs, around 15% will be lost before implantation begins. Of those which begin to implant, only about half will implant successfully. Of the half which do implant successfully (as shown by detectable HCG in the woman’s urine), between one third and one half will be lost at the time of the menses. Overall, therefore, around 75% of all conceptions are followed by an apparently normal period. ^1^” ^m^

In the original witness statement, this passage includes a citation (Footnote 1, shown above) to a short (approx. 280 words) article ^7^ published by Drife over 18 years previously in the *British Medical Journal* ^n^. This brief article, entitled *What proportion of pregnancies are spontaneously aborted?* contains four citations. The first two are reviews by Short ^8^ and Schlesselman ^9^ that draw their quantitative conclusions ultimately from the same primary sources: namely, the unique anatomical studies of Arthur Hertig ^10^, and French & Bierman’s observational study of 3,197 pregnancies in Kauai in the 1950s ^11^. Henri Leridon, a renowned epidemiologist, used these same data to produce a complete life table for intra-uterine mortality in 1977 ^6^. Leridon’s review has been widely cited (although not directly by Short) and his life table is reproduced as Table II in Schlesselman’s review. Strangely, despite using the same sources, Short concludes that “only about 47% of conceptions will result in a full-term live birth” ^8^ whereas Leridon’s estimate is 37% (31/84) ^6^. Drife’s third citation is a brief article in *The Lancet* by Roberts & Lowe, which concludes that 78% of all fertilised eggs perish before birth ^12^. The fourth citation is a report by Miller *et al*. (1980) of a prospective study of 197 women ^13^, estimating the loss of implanted embryos before clinical recognition of pregnancy, i.e., embryo loss between 1 and 2 weeks post-fertilisation.

These sources have been critically reviewed ^5^. In summary: (1) Hertig’s quantitative estimates are highly imprecise; (2) French & Bierman’s study provides no relevant data regarding embryo loss during the two weeks after fertilisation; (3) Roberts & Lowe’s conclusions are derived from speculative arithmetic and have no practical quantitative value; (4) when compared to subsequent studies, Miller’s estimate of 43% loss from implantation to birth (cited by Drife) is almost certainly an overestimate.

The quantitative accounts in *WSJD* and the brief *BMJ* article partially follow Leridon (see Figure 1). The 85% fertilisation rate matches Leridon’s 84%, and derives ultimately from Hertig ^14^. The pre-implantation loss of 15% is also similar to Leridon’s. There are however, some inconsistencies between Drife’s two accounts. For example, in the *BMJ* article he closely follows Leridon in stating that 15 fertilised ova fail to implant (i.e., 15/85 = 18%), but reports this as 15% (i.e., 15/100 = 15%) in *WSJD*. Furthermore, the conclusion in his witness statement that “75% of all conceptions are followed by an apparently normal period” ^o^ does not match the claim published in the *BMJ* that “the proportion of pregnancies lost after conception is 76%” ^p^, and substantially differs from Leridon’s estimate for embryo loss before a normal period of 50%. This is principally because of the addition of an extra stage of loss between implantation and the first missed period ( Figure 1). Hence, Drife overstated (perhaps inadvertently) the extent of early embryo mortality in his witness statement compared to both his own published article and the most authoritative source on which he relied.

In his 1983 *BMJ* article, Drife cited Miller *et al.* (1980) who probably exaggerate early pregnancy loss ^5^. However, in 2001, the date of the witness statement, at least eight relevant studies on early embryo mortality had been published ^5^, including a seminal work by Wilcox *et al*. (1988) ^15^. An expert witness might have been expected to refer to some or all of these works.

This consideration of Drife’s expert testimony highlights four key points:

1. Drife had written and published little on the subject of human embryo mortality.
2. Published claims regarding human embryo mortality are scant, confusing and contradictory.
3. Drife’s claim that 15% of embryos are lost in the first week after fertilisation before implantation is drawn from Leridon’s widely known and respected review of embryo mortality.
4. Drife’s claim that 75% of all embryos are lost before an apparently normal period is an exaggeration that contradicts both Leridon’s account and his own published article!

### B. Professor Nigel Andrew Brown

Munby J. quotes from Professor Brown’s witness statement ( *WSNB*) only once: “It is striking that the usual fate of the fertilized egg is to die” ^q^. Alone, this statement lacks quantitative rigour, since all fertilised eggs eventually die, the substantive issues being when and how many. However, immediately following this, in his witness statement, Professor Brown continues:

“The proportion of fertilized eggs that produce a live full-term baby (in the absence of contraceptive measures) is not known precisely, but is probably only 40% ^1^. The other 60% die, at all stages from fertilization to late pregnancy. Perhaps 20% or so do not implant in the uterus; there are no systemic signs that fertilization has occurred, and the woman is unaware. The next common stage of conceptal death is soon after implantation, when the consequence can be a heavier than usual menstrual flow, perhaps somewhat delayed, which can be noticeable.” ^r^

This passage, from a section entitled “The Incidence of Death of Fertilised Eggs”, contains all the quantitative information on human embryo loss in *WSNB*. 20% loss prior to implantation is similar to the value of 15% given by Drife. Total loss of 60% from fertilisation to birth is close to Leridon’s estimate of 63%. However, the study ^16^ providing the source for the 40% has been misinterpreted: the 60% ^s^ loss in Edmonds *et al.* (1982) actually indicates embryo loss from implantation and not from fertilisation as stated by Brown. Unfortunately, the data in Edmonds *et al.* are likely to be substantially biased owing to sup-optimal experimental design and methodology ^5^. As noted above, more and better studies had been published by 2001 and all reported substantially lower estimates of post-implantation embryo loss.

Thus, in summary:

1. As already noted, available scientific evidence on human embryo mortality is easily misread.
2. Brown’s ball-park figure for mortality from fertilisation to birth of 60%, despite being based on a misreading of a technically biased study, is close to Leridon’s estimate of 63%. Both of these estimates are somewhat lower than Drife’s estimate of 75% loss before an apparently normal period.
3. Brown’s estimate of 20% for pre-implantation loss is close to Drife’s (and Leridon’s) estimate of approximately 15%.

### C. Professor Peter Riven Braude

The interpretation of the evidence submitted by Professor Peter Braude is at the heart of the scientific misunderstanding in this case. Munby J. deliberately emphasises the extent of embryo loss as follows:

“There is one other aspect of this medical evidence which perhaps requires emphasis. This is summarised by Professor Braude in the proposition that “Fertilisation does not usually result in the development of an embryo” and by Professor Brown in the statement “It is striking that the usual fate of the fertilized human egg is to die.” According to Professor Braude not much more than 25% of successfully fertilised eggs reach the blastocyst stage of development and “Even once implanted the failure rate is prodigious”, for fewer than 15% of fertilised eggs will result in a birth.” ^t^

Professor Braude submitted to the court as evidence both a witness statement ( *WSPB*) and a book chapter, entitled *The Embryo in Contemporary Medical Science* ^17^, listed as Exhibit *PB/2*, jointly written and published in 1990 by him and a colleague, Professor Martin Johnson ^u^. The values emphasised by Munby J. in paragraph 129 of his judgment are from this book chapter. Braude does not use these values in his witness statement but merely states that “It is to be noted that of the eggs that are successfully fertilised, a large number do not eventually become implanted in the uterine wall.” ^v^ He provides no citation for this claim.

Unlike Drife and Brown, Braude cites the best available study of early pregnancy loss at that time, Wilcox *et al.* (1988) ^15^, stating that “nearly one quarter (22%; 43/198) of women attempting pregnancy, showed a positive hCG but did not continue to miss their menstrual period or continue with a clinical pregnancy.” ^w^ Braude also refers to another study, albeit without an explicit citation; however, it is clear from the context that the study is Ellish *et al.* (1996) ^18^. He reports a post-implantation embryo loss in this study of “between 11% and 27%” ^x^ prior to the first missed period. These values are from Table VI of Ellish *et al.* (1996), and are consistent with the equivalent value (22%) from Wilcox *et al.* (1988) and many other studies ^5^. However, it is important to note that neither Wilcox *et al.* (1988), Ellish *et al.* (1996), nor the two similar studies cited by Drife (Miller *et al.*, 1980) or Brown (Edmonds *et al.*, 1982) contains any data on embryo loss between fertilisation and the onset of implantation.

Exhibit *PB/2* is an extract from a book that examines the human embryo from historical, legal and cultural perspectives. It is a scientific chapter ^17^ and its purpose is stated clearly in the introduction: “It is our intention here to summarize as simply as possible some of our current knowledge about human early development which can serve as a basis for informed discussion.” It is a description of the formation of germ cells (sperm and ova), fertilisation, and the development and growth of the embryo up until birth. The principal focus is on the period from fertilisation up until the fetal stage, thereby reflecting the subject matter of the book as a whole. The account is very useful.

The values used by Munby J. in paragraph 129 are in one section of this chapter. Following Munby J.’s example, I shall quote the paragraph at length, underlining those phrases reproduced verbatim in his judgment:

*“ Fertilization does not usually result in the development of an embryo*. From our knowledge of human development *in vitro* and those limited studies of early human development *in vivo*, it seems that not much more than 25 per cent of successfully fertilized eggs reach the blastocyst stage of development. ^16^ Even once implanted the failure rate is prodigious. A recent study has suggested that 22 per cent of very early pregnancies which can be detected by raised blood levels of human chorionic gonadotrophin (hCG; the hormone produced by the implanting trophectoderm) will fail. ^17^ This group does not include those pregnancies that fail before the hCG can be produced and thus go undetected. In addition, a further 12–15 percent of clinically recognized pregnancies fail within the first 4 months of pregnancy. ^18^ In all, fewer than 15 per cent of fertilized eggs will result in a birth.” ^y^

This passage contains several quantitative claims. A 12–15% clinical loss is a credible estimate ^5^. The reference to 22% is from Wilcox *et al.* (1988) and is followed by the important point, highlighted above, that those data do not include pregnancies that fail after fertilisation but before implantation ^z^. In other words, that study, and by extension all studies that monitor pregnancy by detection of elevated hCG, cannot inform us about embryo mortality rates in the week after fertilisation but before implantation, that is, during the period that the MAP is typically used. The remaining two values in this passage, both reproduced in the judgment, namely, 25% survival from fertilisation to blastocyst, and fewer than 15% survival from fertilisation to birth, require further inspection.

Munby J. knew that the blastocyst stage is reached prior to the commencement of implantation as indicated by his account of the physiology ^aa^. Table 1 in Exhibit *PB/2* ^17^, which Munby J. appears to have read in detail, also makes it clear that the blastocyst stage is reached at 5 days, before implantation at 7 days. Hence, it is clear that Braude & Johnson’s claim that “not much more than 25% of successfully fertilized eggs reach the blastocyst stage of development” is in stark contrast to that of Drife and Brown, that 15% or 20% of embryos are lost before implantation. According to one, only 25% survive, and according to the others, 80–85% survive to the blastocyst stage. This difference is substantial ( Figure 1) and the inconsistency invites scrutiny. Did Munby J. notice this discrepancy in estimates of the same phenomenon occurring within a few paragraphs of his own judgment? Is it by chance that he chose to emphasise the one value from all the submitted evidence that maximised and exaggerated the extent of embryo mortality prior to implantation, precisely the time when the MAP is intended for use? It may be stated that if Munby J. believed that this value referred to the mortality of embryos *in vivo* under natural conditions, conditions under which the MAP is typically used, he was unequivocally wrong.

The basis for his error is not difficult to identify and will be clarified after considering the evidence provided by the fourth expert witness.

### D. Dr John McLean

Dr McLean provided the longest written statement ( *WSJM1*) of all the expert witnesses ( Table 1). Specifically, his statement included a section entitled “Early Embryo Loss” ^bb^ comprising 740 words and 10 scientific references. (McLean provided a second witness statement ( *WSJM2*), which addressed the issue of the dating of the commencement of pregnancy. It contains no additional information on human embryo mortality.) McLean distinguishes between different categories of study on embryo loss: those that, in principle, provide information on embryo loss before implantation ^cc^, and those that provide information on loss only after implantation has commenced ^dd^. In the first category, McLean discusses in some detail the unique studies of Hertig previously mentioned ^10^. As noted with Drife and Brown, there are minor errors in McLean’s account. For example, 42 is not the maximum age of the 210 women enrolled in the study, but the maximum age of those 34 women from whom fertilised ova were recovered. More importantly, he gives three strikingly variant summary values for early embryo loss derived from Hertig: 29%, 35% and 78%.

McLean’s first value of 29% is his own calculation derived from the 10 abnormal embryos found by Hertig out of all 34 embryos recovered (10/34 = 0.29). Hertig does not use this value nor such a calculation. Making sense of Hertig’s data and calculations is not straightforward, although it is clear that only 8 of the 34 embryos recovered by Hertig were at a pre-implantation stage. An attempt to clarify his calculations has recently been published ^5^, and argues that Hertig’s data and analytical logic indicate that 50% ^ee^ of fertilised eggs would perish up to the time of a missed menstrual period, and that 30% would perish between fertilisation and implantation ^ff^. Leridon’s equivalent values are 50% and 18%. McLean’s second value, 35%, comes from a re-interpretation of Hertig’s data by James (1970) ^19^. However, James’ value of 35% refers to loss of all fertilised eggs before the first missed period and not just before implantation. Hence, James’ estimate is less than both Hertig’s and Leridon’s for the first two weeks after fertilisation, and he concludes that “49% of all zygotes perish naturally between fertilization and confinement” ^gg^. McLean’s third value of 78% is from Roberts & Lowe’s *Lancet* article ^12^. Roberts & Lowe cite Hertig’s work in support of their quantitative argument although it is unclear how it meaningfully informs their analysis ^hh^. Furthermore, Roberts & Lowe’s 78% estimate is not for early pregnancy loss, but for embryo loss from fertilisation to birth. Though widely cited, it is both exaggerated and unsupported by evidence.

In giving the impression that Roberts & Lowe’s estimate is derived from Hertig’s data, McLean’s account is inaccurate and unhelpful. Nevertheless, such a large numerical variance might alert an attentive reader to the difficulties associated with detecting pre-implantation human embryos, an issue McLean explicitly discusses ^ii^. In paragraphs 23–25 of *WSJM1*, he considers two putative biological markers, early pregnancy factor (EPF) and embryo-derived platelet activating factor (EDPAF), that had been proposed to be released within 24 hours of fertilisation. He discusses the possibility that detection of EDPAF might provide insight into the fate of embryos in the first week ^jj^; however, he offers no quantitative estimates from such investigations. Since 2001, little work has been published on EPF or EDPAF and any initial promise they may have had for detecting pre-implantation embryos has long since faded ^kk^. Munby J. was correct in stating that “The test for hCG represents the first reliable opportunity to identify the existence of an embryo ^ll^.”

*WSJM1* also contains results from three studies that used the detection of hCG to quantify embryo loss between implantation and a clinical diagnosis of pregnancy. The estimates were 33% ^13^, 57% ^16^ and 8% ^20^ early pregnancy loss. Once again, the divergence in these values is striking and may make a thoughtful reader query their reliability. The high variance in the results from these three studies is likely to be due to limitations in experimental reagents and study design ^5^. Given the length and detail of his statement, it is surprising that McLean does not mention Wilcox *et al.* (1988), the first of several studies to address these limitations. Nevertheless, McLean does make it clear that even these studies cannot, in principle, provide information on the fate of the pre-implantation embryo.

McLean also makes the important point that the extent of early embryo loss is not only a matter of biological interest, but “has acquired political significance with regard to legislation on human embryo experimentation and the use of emergency hormonal contraception” ^mm^. Arguably, Munby J.’s emphasis on this aspect of the scientific evidence lends credence to this point. McLean continues: “It is therefore important to obtain as accurate an estimate as is possible for the occurrence of early human embryo loss.” As if to reinforce this point, he cited a study by Walker *et al.* (1988) ^21^ that reported no losses of biochemical pregnancies ^nn^ in 75 cycles and concluded that the extent of early pregnancy loss may have been “substantially overestimated.” ^oo^ This estimate of 0% loss is extreme, and a reasoned response from Wilcox argued that the results of their two studies were not necessarily inconsistent ^22^. However, the message is clear: quantification of early embryonic loss generates confusing and highly variable results and it therefore behoves a cautious reader concerned with factual accuracy to scrutinise specific quantitative claims with care.

In emphasising that “not much more than 25% of successfully fertilised eggs reach the blastocyst stage”, a value that is quantitatively contradicted by the evidence provided by Drife, Brown and McLean ( Figure 1), Munby J. reveals that he did not properly understand the significance of the expert witness evidence provided to the court on this matter. Furthermore, he cannot have examined that particular claim with due care, for if he had done, he would have discovered why the value was so low and why it was of no relevance to the case.

## Munby J.’s error

In paragraph 129 of Munby J.’s judgment there are two quantitative claims, both attributed to Professor Braude, that invite close scrutiny: (1) “not much more than 25% of successfully fertilised eggs reach the blastocyst stage of development”, and (2) “fewer than 15% of fertilised eggs will result in a birth”. Although Munby J. does not place these statements in quotation marks, they are taken directly from the book chapter submitted by Braude as evidence (Exhibit *PB/2*) ^17^. In this chapter, the 25% claim is supported by a citation to a review on the early development of human embryos *in vitro* co-written by Dr Virginia Bolton and Professor Braude ^23^. A “<15%” claim is found in the same review.

Bolton & Braude’s review of the development of the preimplantation human embryo *in vitro* represents IVF as “a remarkably inefficient therapeutic procedure” and explores biological and technical reasons for the “unacceptably high rate of embryonic loss” associated with IVF treatment. Within this context they describe the work of Fehilly *et al.* (1985) ^24^ who…

“…attempted to culture human embryos surplus to those required for replacement during therapeutic IVF cycles to the blastocyst stage for cryopreservation. Of 784 pronucleate embryos, 75% (585) were able to develop to the five- to eight-cell stage *in vitro*; only 34% (197) of these progressed in culture to form expanded blastocysts.”

197 expanded blastocysts developing from 784 embryos is 25.1%, i.e., “not much more than 25%”.

Fehilly *et al.* (1985) conducted their study primarily to determine whether embryos frozen at the cleaving stage (day 3–4 after fertilisation) or at the blastocyst stage (day 5–6 after fertilisation) would be more effective at producing subsequent pregnancies after embryo thawing and transfer into the womb. Based on the data described above, with regard to embryo development to the blastocyst stage, they commented that “only one quarter of them expanded in vitro”. This data is the source for Munby J.’s claim that not much more than 25% of fertilised eggs reach the blastocyst stage.

In the following paragraph in their review, Bolton & Braude offer one reason for the low survival rate of the *in vitro* embryos: “Suboptimal culture conditions are undoubtedly responsible for a proportion of this embryonic failure” ^23^. Elsewhere, Braude reports that “Experiments in our laboratories have suggested that the in vitro handling of oocytes can produce chromosomal aberrations at alarmingly high frequencies” ^25^ and strikingly, that “When Bob Edwards and indeed my own group were researching these early stages, blastocyst culture was awful (about 15% of embryos made it to that stage)” ^26^. More recently, Dr Bolton has repeated that “Embryo culture conditions *in vitro* are likely to be suboptimal compared with those *in vivo*” ^27^. Put simply, human embryos created by fertilisation *in vitro* did not, and do not fare well. Hence, the use of *in vitro* data to define the fate of natural ** embryos *in vivo* is both biologically and quantitatively risky ^5^.

Munby J.’s second quantitative claim, that “fewer than 15% of fertilised eggs will result in a birth”, also comes from Bolton & Braude’s review via Braude & Johnson’s chapter. A further quotation from p. 93 makes the point:

“In fact, IVF represents a remarkably inefficient therapeutic procedure. Although fertilization can now be achieved with consistent success *in vitro*, the success rate of ongoing pregnancies is much lower … If an average is taken from the longest established IVF units, it can be seen that <15% of all embryos that are replaced will result in a clinical pregnancy (Table I).” ^23^

Table I in Bolton & Braude provides a quantitative summary of the success of embryo replacement using data from seven clinical IVF units. The data are sub-divided according to whether 1, 2 or 3 embryos were replaced (i.e., transferred) into the womb of the woman undergoing treatment. The clinical pregnancy rate among the patients thus treated is reported as a percentage of embryos replaced as 12.7%, 12.1% and 9.6% respectively. These values are somewhat lower than 15% and clearly refer to clinical pregnancies. Nevertheless, this IVF data appears to be the source for the judicial claim that “fewer than 15% of fertilised eggs will result in a birth”.

There are only two other cited studies in the key paragraph of Braude & Johnson’s chapter (Exhibit *PB/2*) ^17^. These are the study by Wilcox *et al.* ^15^ and a paper by Professor Lesley Regan ^28^. Wilcox *et al.* conclude that “The total rate of pregnancy loss after implantation, including clinically recognized spontaneous abortions, was 31 percent” and a re-analysis of that data suggests that approximately 50% of the fertilised eggs in that study may have been lost up to birth ^4^. Regan’s paper addresses the incidence of spontaneous abortion of clinical pregnancies with a focus on recurrent abortion. It contains preliminary findings from a prospective study of pregnancy loss and, interestingly, concludes that the “overall incidence of spontaneous abortion in this prospective study is considerably lower than those reported in previous studies (10.3% overall; 5.6% for primigravidae)” ^28^. Therefore, neither Wilcox *et al.* (1988) nor Regan (1987) is a credible source for Munby J.’s claim that “fewer than 15% of fertilised eggs will result in a birth”.

It is clear therefore that the two values emphasised by Munby J. in his judgment referred firstly, to embryo mortality *in vitro*, and secondly, to the survival of IVF embryos following their transfer into women as part of fertility treatment. It is surely reasonable to suppose that the context for the use of the MAP, subject to the judicial review, was not *in vitro* embryos in a laboratory or women undergoing fertility treatment, but rather naturally conceived embryos and women at risk of pregnancy. The values emphasised by Munby J. have no bearing on the case at all.

## An understandable error?

It is charitable to assume that Munby J. did not realise that the figures he emphasised were biologically misrepresentative and therefore irrelevant to the case. Nevertheless, are there any reasons to believe that he could have spotted and avoided this error?

There are clues in Braude & Johnson’s chapter that might alert an attentive reader to the potentially misleading nature of some of its quantitative claims. In the introduction, they explicitly state that their knowledge of human development was drawn from various sources, including “studies of live preimplantation pre-embryos *in vitro* as part of therapeutic infertility programmes”, and in the critical paragraph from which Munby J. quotes, they preface their remarks with the phrase: “From our knowledge of human development *in vitro* and those limited studies of early human development *in vivo*”. Unfortunately, their subsequent commentary does not make it clear which of the evidence they report is from *in vivo*, and which from *in vitro* studies. Scrutiny of the references, as noted above, is revealing. Since Braude & Johnson aimed “to summarize as simply as possible some of our current knowledge about human early development”, it is perhaps understandable that they glossed over such detail. Consequently, a non-expert reader may be forgiven for concluding that their account related to normal early human development under natural conditions, particularly since no indication is given in the chapter that there are any substantive differences between *in vitro* and *in vivo* embryonic development.

It is unlikely that Braude & Johnson wrote the chapter, published in 1990, with a view to it being submitted as expert witness testimony in a judicial review in 2001, let alone that it should be directly quoted by a judge. Perhaps it would have been wise only to submit a contemporaneous witness statement addressing matters of direct relevance to the case. It would be harsh to even hint that, in submitting the chapter as expert witness evidence, Braude intended to mislead the judge on this particular matter, and it may be asking too much to expect even a learned judge to see through such a tangle of scientific evidence. Nevertheless, irrespective of whether witness, judge or both were culpable, the outcome is clear: Munby J. misjudged the extent of human embryo mortality.

Munby J. had more evidence available to him than just Braude & Johnson’s chapter ( Table 1). The emphasis he placed on human embryonic mortality must therefore be read in the context of that further expert witness testimony. Arguably, the contradictions between the various statements (see Figure 1) should have alerted him to these issues. Other than to acknowledge its existence, Munby J. makes no reference to McLean’s detailed and lengthy statement, prepared on behalf of the Claimant. This is regrettable, since the statement offers credible warnings about the relevance and reliability of estimates of embryo mortality in the scientific literature. The similar numerical estimates for pre-implantation loss offered independently by both Drife and Brown are in stark contrast to the one he chose to emphasise from Braude & Johnson’s chapter. Furthermore, in selectively weaving quotations from the witness testimonies, Munby J. gives the misleading impression that Braude and Brown were in agreement about the extent of early embryo mortality, despite evidence to the contrary.

One is left wondering whether Munby J. realised what he was doing.

## Conclusion

Braude & Johnson’s chapter was written with more than just biology in mind: the critical paragraph is in a section headed “ *Ethics and the biology of pre-embryos*” and the conclusion touches on religious, ethical and regulatory issues. It thus appears that there was an ethical and, by extension, a legislative agenda underlying this chapter. This agenda is more explicit in a magazine article ^29^ cited by Braude & Johnson.

Natural human embryo mortality has often been linked to the ethical status of human embryos. For example, in their brief article, Roberts & Lowe state that “If Nature resorts to abortion … by discarding as many as 3 in every 4 conceptions, it will be difficult for anti-abortionists to oppose abortion on moral and ethical grounds.” ^12^ Ronald Green, Professor Emeritus of Religion at Dartmouth College, points out, incorrectly, that “between two-thirds and three-quarters of all fertilized eggs do not go on to implant in the womb” and asks: “In view of this high rate of embryonic loss, do we truly want to bestow much moral significance on an entity with which nature is so wasteful?” ^30^ A report of the Ethics Committee of the Royal College of Obstetricians and Gynaecologists in 1983 states: “Knowing as we do that in the natural process large numbers of fertilised ova are lost before implantation, it is morally unconvincing to claim absolute inviolability for an organism with which nature itself is so prodigal” ^31^. This link has been considered by many others ^32– 34^. Thus, McLean’s assertion, in evidence, that early embryo loss is not only of biological interest but also of political and legislative significance ^pp^, was clearly correct. How specific estimates of embryo mortality inform an ethical calculus is, perhaps, not so clear. Nevertheless, for those who consider it germane, McLean’s exhortation that “It is therefore important to obtain as accurate an estimate as is possible for the occurrence of early human embryo loss”, must surely be correct too.

Did the quantitative bias in Munby J.’s description of embryo mortality have a significant influence on his legal judgment? If not, why then, one may ask, in such a “very long” judgment, would he deliberately choose to emphasise this particular point? What purpose might such an observation serve? Perhaps these are questions for other, legal minds. However, the biology is a different matter. Judgments carry weight and can influence opinion. Even on biological matters, legal scholars may rely upon the sayings of learned judges rather than scientific evidence.

In the first four editions of her popular undergraduate textbook ^35– 38^, Emily Jackson discusses Munby J.’s judgment and comments on embryo loss: “Approximately 75 per cent of all naturally fertilized eggs will be lost before the woman’s next period, and it would be counterintuitive to describe these losses as miscarriages” ^qq^. Although she does not offer the judgment as her source, her words closely reflect Drife’s evidence, repeated verbatim in the judgment ^rr^. Her addition of the word “naturally” makes explicit the implied, and incorrect, sense in the judgment. It is notable that in the most recent (2019) edition of her book ^39^ the explicit 75% claim is omitted: “The majority of naturally fertilized eggs will be lost before the woman’s next period, and it would be counterintuitive to describe these losses as miscarriages.” ^ss^

Another legal scholar, Jonathan Herring, in all eight editions of his textbook ^40– 47^, quotes directly from paragraph 129 of Munby J.’s judgment in order to outline an ethical argument:

“Those who disagree with the argument that personhood begins at conception … could also make the following argument: ‘[It] is striking that the usual fate of the fertilized human egg is to die.’ ^†^ It has been estimated that fewer than 15 per cent of fertilized eggs will result in a birth. ^§^” ^tt^

The first reference ( ^†^) correctly attributes these words to Professor Brown, as found in the judgment. Strangely, the second reference ( ^§^) is to an article by Professor John Harris on assisted reproductive technological blunders ^48^ that has no bearing on the issue. Clearly, it is a direct quotation from Braude & Johnson (1990) ^17^ and paragraph 129 of Munby J.’s judgment.

It is unfortunate that these biological errors have found their way into standard legal textbooks. Irrespective of the philosophical merits of arguments such as those outlined above, most would agree that in order to arrive at defensible ethical and legal conclusions it is necessary to begin with reliable and relevant evidence. It would be helpful if Munby J. were to clarify whether he realised that the numerical estimates he emphasised in paragraph 129 of his judgment were derived from *in vitro* circumstances, and were therefore not representative of natural *in vivo* situations. It would also be instructive to know whether Munby J. still believes that “not much more than 25% of successfully fertilised eggs reach the blastocyst stage of development”. If the answers to these queries are negative, a clarification from him regarding the biology would be welcome, in the hope that the widely held, unsubstantiated and excessively pessimistic view of natural human embryo survival may, little by little, be replaced with one that is more closely based on available, relevant, scientific evidence ^5^.

## Data availability

### Underlying data

Apollo - University of Cambridge Repository: UnderlyingData_MisjudgingEarlyEmbryoMortality_Redacted, https://doi.org/10.17863/CAM.53696 ^3^

This dataset consists of transcripts of scientific witness statements submitted as evidence in *R (on the application of Smeaton) v Secretary of State for Health* [2002] EWHC 610 (admin) (18 April 2002) (Case No: CO/928/01). Some content has been redacted, including personal addresses.

Copyright (“all rights reserved”) for the content of the witness statements remains with the authors of the statements.

Photocopies of original expert witness statements were obtained from the archives of the Claimant. A full unredacted (except for personal addresses) transcript and copies of the statements are available from the author on request.

## Notes

^a^ Paragraphs from the judgment (and witness statements) are referenced in footnotes as follows: *Munby* [1]. The full judgment is available here: http://www.bailii.org/ew/cases/EWHC/Admin/2002/610.html

^b^ *Munby* [346].

^c^ *Munby* [2].

^d^ The Civil Procedure Rules, r.32.12(2)(c)

^e^ The Civil Procedure Rules, r.31.22(1)(a)

^f^ *Munby* [32].

^g^ *Munby* [125] & [191].

^h^ *Munby* [32].

^i^ *Munby* [126].

^j^ *Munby* [126(iii)].

^k^ *Munby* [126(ix)].

^l^ *Munby* [131-5] & [137]; *WSJD* [3-4], [8], [11-12] & [16].

^m^ *Munby* [134]; *WSJD* [8]. Footnote 1 (shown at the end of the quotation) is found in *WSJD* but omitted from the judgment, and reads as follows: “1 Drife, JO. British Medical Journal 1983; 286:294.”

^n^ The *BMJ* has no record of whether this response to a reader question (or similar short items) would have been peer-reviewed in 1983 (personal email correspondence from *BMJ* editorial office, 23 ^rd^ June 2020).

^o^ *WSJD* [8]. This claim only makes sense if his estimate of post-implantation loss of ‘between one third and one half’ is interpreted as 42% (average of 33% and 50%). However, in his *BMJ* piece, Drife states that out of 36 women with detectable hCG, only 24 will miss a period, indicating a loss at this stage of 12/36 = 33%. Thus, his estimate of post-implantation loss is inflated in *WSJD* compared to the *BMJ*.

^p^ The *BMJ* figure incorporates an estimate (‘between 10% and 30%’) for the rate of pregnancy loss after a woman knows she is pregnant. To be internally consistent in the *BMJ* piece, a clinical pregnancy failure rate of 15% must be used. Applying this estimate to *WSJD* results in a total loss between fertilisation and birth of 79%.

^q^ *Munby* [129]; *WSNB* [22].

^r^ *WSNB* [22]. Footnote 1 in this passage reads as follows: “Edmonds D.K., Lindsay K.S., Miller J.F., Williamson E., Wood P.J. Early Embryonic Mortality in Women, Fertility and Sterility 1982 Vol 38 447–453”.

^s^ The precise value reported by Edmonds *et al.* (1982) was 61.9%.

^t^ *Munby* [129].

^u^ It is both likely and appropriate that Munby J. would have regarded Professor Johnson also as a “very eminent medical expert”.

^v^ *WSPB* [8].

^w^ *WSPB* [15]. Braude’s description of this data is slightly inaccurate. The study followed 221 women who were attempting to get pregnant, and the value of 22% (43/198) actually refers to the number of hCG positive menstrual cycles (198 out of 707 monitored cycles) that did **not** manifest as a clinical pregnancy, i.e., 43. 22% therefore refers to embryo loss from the onset of implantation (as indicated by the positive hCG test) up to, but not including, clinical diagnosis (as indicated by an absent menstrual period or subsequent positive pregnancy test).

^x^ *WSPB* [15].

^y^ Braude & Johnson (1990), p. 218. The passage contains three endnotes as follows:

Endnote 16 reads: “V. N. Bolton, and P. R. Braude, in A. McLaren and G. Siracusa, eds, *Current Topics in Developmental Biology*, Vol 23, *Recent advances in mammalian development*, Academic Press, 1987, pp. 93-114.”

Endnote 17 reads: “A. J. Wilcox, C. R. Weinberg, J. F. O’Connor, D. D. Baird, J. P. Schlatterer, R. E. Canfield, E. G. Armstrong and B. C. Nisula, Incidence of early loss of pregnancy’, *New Eng. J. Med.*, 319, 1988, 189-94.”

Endnote 18 reads: “L. Regan ‘A prospective study of spontaneous abortion’, in: R. W. Beard and F. Sharp, eds, *Early Pregnancy Loss*, 18th Study group of the Royal College of Obstetricians and Gynaecologists, Springer-Verlag, 1987, pp. 23-37.”

^z^ Given the matter under consideration in the judicial review, it is notable that, in this passage, Braude & Johnson clearly use the word ‘pregnancies’ to refer to women carrying a fertilised egg before implantation.

^aa^ *Munby* [126(vii)] & [126(viii)].

^bb^ *WSJM1* [30–34].

^cc^ *WSJM1* [31] & [33].

^dd^ *WSJM1* [32].

^ee^ This value of 50% ultimately derives from the 8 pre-implantation embryos, of which 4 were abnormal and therefore assumed to be destined to perish before the end of the first 2 weeks after fertilisation. It is therefore based on a very small sample size.

^ff^ It is a numerical coincidence that McLean’s value of 29% (10/34) is so close to this value of 30%.

^gg^ A zygote is the newly fertilised ovum: a one-cell embryo. Confinement is the time of childbirth.

^hh^ For example, Roberts & Lowe use Hertig’s work as a source for their claim that coitus at the time of ovulation results in a fertilisation rate of 50%. This contrasts with Hertig’s own conclusion, followed by Leridon, that ‘when conditions are optimal about 15 per cent of oocytes fail to become fertilized’ (Hertig, 1967).

^ii^ *WSJM1* [33].

^jj^ *WSJM1* [33].

^kk^ A PubMed ( https://www.ncbi.nlm.nih.gov/pubmed) search on <"early pregnancy factor"[All Fields] NOT Review[ptyp]> performed on 3 ^rd^ June 2020 identified 142 articles published between 1977 and 2000, and 35 from 2001 to the present day. A search on <“embryo derived platelet activating factor"[All Fields]> identified only 26 articles, published between 1985 and 2004. Only two of these were published after 1992.

^ll^ *Munby* [126(ix)].

^mm^ *WSJM1* [34].

^nn^ “Biochemical pregnancy”: this term is sometimes used to refer to pregnancies that are detected solely by elevated hCG rather than by clinical observations such as a missed menstrual period.

^oo^ This phrase is found both in *WSJM1* [34] and the abstract of Walker *et al.* (1988).

^pp^ *WSJM1* [34].

^qq^ Jackson, E., *Medical Law: Text, Cases, and Materials*. 1 ^st^ ed. (2006), p. 624; 2 ^nd^ ed. (2010), p. 693; 3 ^rd^ ed. (2013), p. 703; 4 ^th^ ed. (2016), p. 737.

^rr^ “…around 75% of all conceptions are followed by an apparently normal period. These losses … are not covered by the term ‘miscarriage’.” from *WSJD* [8] and *Munby* [134].

^ss^ Jackson, E., *Medical Law: Text, Cases, and Materials*. 5 ^th^ ed. (2019), p. 770.

^tt^ Herring, J., *Medical Law and Ethics*. 1 ^st^ ed. (2006), p. 250; 2 ^nd^ ed. (2008), p. 285; 3 ^rd^ ed. (2010), p. 310; 4 ^th^ ed. (2012), p. 318; 5 ^th^ ed. (2014), p. 320; 6 ^th^ ed. (2016), p. 332; 7 ^th^ ed. (2018), p. 331; 8 ^th^ ed. (2020), p. 374.
