# Supplementary material for: Misjudging early embryo mortality in natural human reproduction
Source: F1000Res. 2020 Jul 14;9:702. [Version 1] doi: 10.12688/f1000research.22655.1 (PMC7670474; doi:10.12688/f1000research.22655.1)
Supplement: Peer review history

# Peer review: Reviewer response for version 1

**DOI:** 10.5256/f1000research.25011.r74206

**Published:** 2020-11-16

**Authors:** Nicanor Austriaco, OP

**Affiliations:** 1 Department of Biology, Providence College, Providence, RI, USA; 2 Department of Biological Sciences, University of Santo Tomas, Manila, Philippines

In this paper, Gavin Jarvis examines the scientific and medical evidence that was presented by expert witness testimony during a judicial review conducted by the Honorable Mr. Justice Munby of the administrative court in 2002 to determine whether supplying the morning after pill (MAP) is comparable to the procuring of a poison for the purpose of an abortion. If so, the claimant argued that the MAP should be regulated in the same way as any other abortion, as required by the Abortion Act 1967. Justice Munby ruled against the claimant, relying heavily on expert witness testimony that claimed that 75% ****of human embryos that are conceived are lost during pregnancy. Jarvis interrogates each of the witness testimonies in great detail and provides a genealogy of each of the scientific and medical claims. He successfully shows that the available data do not support the conclusion that human embryo mortality is high at 75%.

 The claims and conclusions made by the author are validated by sound reasoning. He provides frequent citations to the primary literature to support his argument that the expert witness testimonies were often inaccurate or inconsistent. As Jarvis himself acknowledges, it would be helpful for someone to determine the impact of his analysis on the law.

 In sum, this essay is a robust and helpful critique of a legal precedent that relied heavily on weak scientific and medical data to claim that human embryo mortality is astronomically high.

Is the topic of the opinion article discussed accurately in the context of the current literature?

Yes

Are arguments sufficiently supported by evidence from the published literature?

Yes

Are all factual statements correct and adequately supported by citations?

Yes

Are the conclusions drawn balanced and justified on the basis of the presented arguments?

Yes

Reviewer Expertise:

Molecular and cellular biology. Bioethics.

I confirm that I have read this submission and believe that I have an appropriate level of expertise to confirm that it is of an acceptable scientific standard.

# Peer review: Reviewer response for version 1

**DOI:** 10.5256/f1000research.25011.r67635

**Published:** 2020-08-11

**Authors:** Maureen L. Condic

**Affiliations:** 1 Department of Neurobiology and Anatomy, School of Medicine, University of Utah, Salt Lake City, UT, USA

The article “ *Misjudging early embryo mortality in natural human reproduction” *considers the evidence presented in a 2002 court case [ *R (on the application of Smeaton) v Secretary of State for Health*], involving the use of Levonelle (the ‘morning after pill’; MAP) as a form of birth control. In this case, it was ruled that MAP does not act to terminate a pregnancy, a conclusion that relied heavily on the assertion that only 25% of successfully fertilized eggs survive to implantation. Here, the scientific and medical evidence in support of this assertion is examined, based on the published literature and the testimony of the four expert witnesses who provided numerical estimates of embryo mortality.  In particular, the figures relied on in the Judicial opinion are derived from studies of embryo survival during IVF, and are therefore irrelevant to the use of MAP as birth control.

 The argument is clearly written and provides ample evidence in support of the author’s conclusion that the case was decided, based in part, on a misinterpretation of the scientific evidence. The author points out the need for a legal analysis to clarify the impact of this error, yet (appropriately) does not offer an opinion on this matter.

 Over all, this is a helpful and clear critique of a seminal case involving human embryo mortality.

Is the topic of the opinion article discussed accurately in the context of the current literature?

Yes

Are arguments sufficiently supported by evidence from the published literature?

Yes

Are all factual statements correct and adequately supported by citations?

Yes

Are the conclusions drawn balanced and justified on the basis of the presented arguments?

Yes

Reviewer Expertise:

Human embryology, Developmental neuroscience, Human stem cell biology, Bioethics

I confirm that I have read this submission and believe that I have an appropriate level of expertise to confirm that it is of an acceptable scientific standard.
